# Effects of acute adult and early-in-life bladder inflammation on bladder neuropeptides in adult female rats

**DOI:** 10.1186/1471-2490-11-18

**Published:** 2011-08-15

**Authors:** Amber D Shaffer, Chelsea L Ball, Meredith T Robbins, Timothy J Ness, Alan Randich

**Affiliations:** 1 Department of Psychology, University of Alabama at Birmingham, CH 415, 1530 3rd Avenue South, Birmingham, AL 35294, USA; 2 Department of Anesthesiology, JT 804, 619 19th Street South, University of Alabama at Birmingham, Birmingham, AL 35294, USA

## Abstract

**Background:**

The purpose of the present study was to determine how acute adult and/or prior early-in life (EIL; P14-P16) exposure to bladder inflammation affects bladder content of calcitonin gene related peptide (CGRP) and substance P (SP). Estrous cycle influences were also studied in the adult-treatment conditions.

**Methods:**

In Experiment 1, intravesical zymosan or isoflurane anesthesia alone was administered to adult female rats. Bladders and serum were collected 24 hours later during each phase of the estrous cycle. In Experiment 2, zymosan or anesthesia alone was administered EIL and as adults, with bladder tissue collection 24 h later.

**Results:**

In general, Experiment 1 showed that bladder content of both CGRP and SP was increased by inflammation. This effect was significant when data were collapsed across all phases of the estrous cycle, but was only significant during proestrus when individual comparisons were made during each phase of estrous. Also, adult bladder inflammation significantly reduced estradiol levels. In Experiment 2, bladder content of CGRP and SP was significantly increased in rats receiving EIL and/or adult inflammation. Bladder weights were also significantly increased by inflammation.

**Conclusions:**

These data indicate that bladder CGRP and SP are maximally increased during the proestrus phase of the estrous cycle in inflamed adult female rats. EIL exposure to bladder inflammation alone can also produce an increase in CGRP and SP lasting into adulthood. Therefore, EIL experience with bladder inflammation may predispose an organism to experience a painful bladder disorder as an adult by increasing primary afferent content of CGRP and/or SP.

## Background

The mechanisms underlying bladder hypersensitivity arising from bladder inflammation have been examined by various investigators through the use of agents such as cyclophosphamide (CYP) [1-4], zymosan [5,6], turpentine [7] and mustard oil [8,9]. These studies indicate that bladder tissue, dorsal root ganglion (DRG), and spinal cord content [1,5,8] of two of the primary neurotransmitters of bladder afferents, calcitonin gene-related peptide (CGRP) and substance P (SP), increase following bladder inflammation and may play a critical role in mediating inflammation-produced bladder hypersensitivity. Functional measures indicate that bladder hypersensitivity is affected by several factors such as the phase of the estrous cycle or the phase of the menstrual cycle in which bladder sensitivity is tested [10,11]. For example, the vigor of the visceromotor reflex (VMR) to urinary bladder distension (UBD) in inflamed female rats is elevated in proestrus relative to controls [6]. In humans, the highest daily "worst pain" scores in women with IC occur during the premenstrual period and urinary symptoms increase in the perimenstrual period [12,13]. In addition to estrous cycle influences, bladder hypersensitivity resulting from bladder inflammation as an adult can be enhanced further by prior early-in-life (EIL; P14-P16) experience with bladder inflammation [14,15], and this experimental paradigm has been advanced as a possible model for studying the etiology of painful bladder syndrome/interstitial cystitis (PBS/IC). However, at the present time, it is not known whether these findings based on functional measures relate in any way to bladder levels of CGRP and SP and so serves as the focus of the present investigations.

The goal of Experiment 1 was to determine whether adult bladder content of CGRP and SP was affected by estrous cycle and either the presence or absence of acute bladder inflammation. The goal of Experiment 2 was to evaluate how an EIL episode with bladder inflammation affected adult bladder content of CGRP and SP either with or without additional adult bladder inflammation.

## Methods

### Animals

In Experiment 1, 12 week old adult female Sprague-Dawley rats were obtained as adults from Harlan. For Experiment 2, timed-pregnant female Sprague-Dawley rats were obtained from Harlan and pups were born and raised in our facilities. All animals were 12-14 weeks old at the time of testing. The light-dark cycle for all rats was 6:00 am - 6:00 pm. All studies were approved by the University of Alabama at Birmingham Animal Care and Use Committee, and conformed to the guidelines of the International Association for the Study of Pain (IASP).

### Lavage

The stage of estrous was determined by daily vaginal lavages beginning at approximately 12 weeks of age. Each rat had to show at least two complete cycles prior to treatment. Estrous determination was as described by Becker et al. [16] where: metestrus- composed mainly of leukocytes with few nucleated and/or cornified cells, diestrus- lacking or nearly lacking the presence of all cells, proestrus- composed mainly of nucleated cells, and estrus- composed mainly of cornified cells.

### ELISA and RIA

At the time of sacrifice, all rats were deeply anesthetized under isoflurane anesthesia (5%). Bladders were removed from all rats and cardiac blood was collected from all rats in the acute, adult conditions. Serum was centrifuged at 1600 rpm for 15 min and stored at -80°C until analyzed. The urinary bladder from each rat was flash-frozen in liquid nitrogen, then stored at -80°C. Bladders were analyzed for SP and CGRP using ELISAs (Phoenix Pharmaceuticals, Burlingame, CA). Progesterone and estradiol levels were determined with radioimmunoassays (RIAs) by the University of Virginia Center for Research in Reproductive Ligand Assay and Analysis Core. However, all group assignments were based on vaginal lavages and not hormone levels.

### Acute Adult inflammation

In Experiment 1, rats were designated into either anesthesia-alone pretreatment or zymosan pretreatment. Zymosan pretreated rats were anesthetized with isoflurane/oxygen (5% induction/2% maintenance) and a 22-gauge angiocatheter was placed into the urinary bladder via the urethra. Intravesical zymosan (1% solution in saline; 0.5 ml) was administered and left to dwell for 30 min before draining and catheter removal. This dose/concentration of zymosan has been previously shown to produce bladder inflammation using the testing parameters of this study [17]. The anesthesia-alone pretreated rats were given only isoflurane anesthesia for 30 min. Each rat was administered s.c. ampicillin after the procedure (200 mg) and awakened. This resulted in two conditions or groups: group A (anesthesia alone) and group Z (zymosan). Anesthesia-alone controls were used in these experiments and not naïve rats because anesthesia involving volatile anesthetics, e.g. halothane versus isoflurane, is known to have markedly different influences on a wide variety of measures of nociception including c-fos expression [18] and first order dorsal horn neurons [19,20] and so they were appropriate controls for the inflamed groups which were also anesthetized with isoflurane.

Group assignment was based on the phase of the estrous cycle the animal was in at the time of testing based on vaginal lavage. We pretreated all rats between 5-6 pm 24 h prior to sacrifice. We did this because progesterone and estrogen have been reported to be closest to their peak levels at 5-6 pm in the proestrus phase [21]. We wanted to maximize the likelihood of observing high levels of these hormones to increase our ability to detect differences between anesthesia-alone and zymosan-treated animals. We also wanted to keep the time of day of both pretreatment and bladder tissue harvesting constant.

### EIL

In Experiment 2, on each day from P14-P16, female rat pups were halothane-anesthetized (5% induction, 1-2% maintenance) via mask, had their urethras swabbed with iodine-povidone solution, and were kept warm with a heating pad. On each day, zymosan-treated groups had a 24-gauge angiocatheter placed transurethrally into the urinary bladder and received intravesical administration of 0.10 ml of zymosan (1% in saline) for 30 min. The anesthesia-alone groups were anesthetized for 30 min but had no intravesical catheter placement or treatment. All rats were given ampicillin (20 mg/kg, s.c.) on each treatment day. An intravesical saline group was not examined as previous studies demonstrated no physiologically important differences between saline-treated groups and those treated with anesthesia alone [15].

As adults, these rats received either anesthesia-alone pretreatment or zymosan pretreatment as described above. This resulted in four conditions or groups: group AA (anesthesia alone EIL and anesthesia alone as adult), group AZ (anesthesia alone EIL and zymosan as adult), group ZA (zymosan EIL and anesthesia as adult), and group ZZ (zymosan EIL and zymosan as adult).

### Statistics

All data are presented as group mean ± standard error of the mean (SEM). Overall ANOVAs were performed and followed by post-hoc t-tests as appropriate (α = 0.05). Holm's correction was used for multiple comparisons.

## Results

### Acute adult inflammation - Anesthesia Alone and Zymosan Conditions

#### CGRP and SP

**Figure 1:**
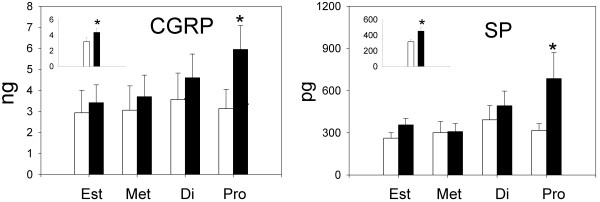
**Figure 1 presents mean group bladder content of CGRP (ng) and SP (pg) during each phase of the estrous cycle in animals treated with either anesthesia (open bars) or zymosan (solid bars) (n = 6-7 rats/group)**. The insets show data for all rats collapsed across phases of the estrous cycle. Error bars depict standard error of the mean (SEM). CGRP and SP were significantly increased by adult zymosan treatment during proestrus as well as when data were collapsed across phases of the estrous cycle (* significantly different from anesthesia control p < 0.05).

Figure 1 presents group mean CGRP and SP levels for anesthesia-alone (open bars) and zymosan-inflamed (solid bars) groups during each phase of estrous and the insets show group mean levels of these peptides collapsed across all phases of estrous. An overall ANOVA revealed a significant effect of intravesical treatment on bladder content of CGRP (F(1,38) = 4.64, p < 0.03) but no significant effect of estrous cycle or estrous cycle × treatment interaction. In general, CGRP content was increased by inflammation and it was significantly greater in the inflamed condition when data from all rats were collapsed across all phases of the estrous cycle as shown in the inset (p = 0.04). Individual estrous phase-matched comparisons between zymosan and anesthesia-alone groups, however, revealed that CGRP levels were significantly greater in zymosan-treated rats only during proestrus (p = 0.04).

An overall ANOVA revealed a significant effect of intravesical treatment group on bladder content of SP (F(1,39) = 6.06, p = 0.02) and a significant effect of estrous cycle (F(3,39) = 2.92, p = 0.05). In general, groups treated with zymosan showed greater levels of SP than estrous-phase matched anesthesia controls and SP content was significantly greater in inflamed rats when data were collapsed across all phases of the estrous cycle as shown in the inset (p = 0.02). Similar to the CGRP data, individual estrous phase-matched comparisons between zymosan and anesthesia-alone groups revealed that SP levels were significantly greater in zymosan-treated rats only during proestrus (p = 0.01). In addition, there was a clear trend for both CGRP and SP levels to increase in zymosan-treated groups as animals progressed through the estrous cycle from estrus to proestrus. In contrast, no such trend was observed in anesthesia-alone groups.

### Bladder Weights

**Figure 2:**
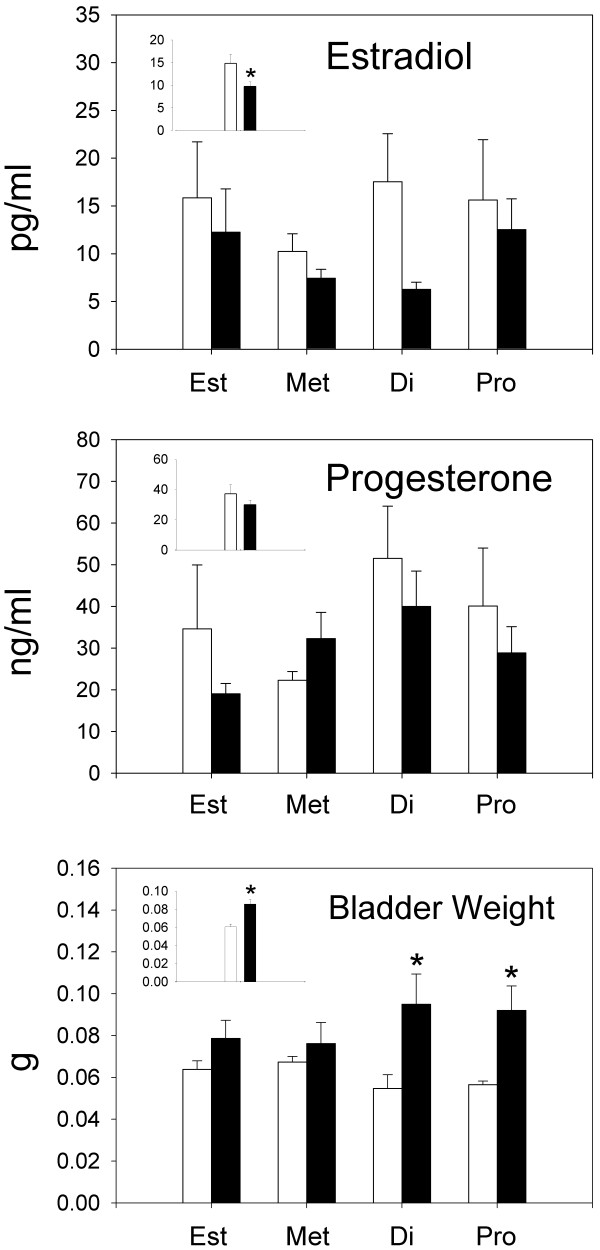
**Group mean estradiol levels (pg/ml), progesterone levels (ng/ml), and bladder weights (g) for each phase of the estrous cycle in animals treated with either anesthesia (open bars) or zymosan (solid bars) (n = 6-7 rats/group)**. Error bars depict standard error of the mean (SEM). Estradiol levels were significantly decreased by adult zymosan treatment when data were collapsed across phases of the estrous cycle. Bladder weights were significantly decreased by adult zymosan treatment during diestrus and proestrus, as well as when data were collapsed across phases of the estrous cycle (* significantly different from anesthesia control p < 0.05).

Bladder weights for Experiment 1 are shown in the bottom panel of Figure 2. Bladder weights did not significantly vary as a function of the phase of the estrous cycle when comparisons were made within either the anesthesia-alone condition (F(3,21) = 1.98; p = 0.14) or the zymosan condition (F(3,20) = 0.55; p = 0.65). However, bladder weights of zymosan-treated rats were significantly greater than those treated with anesthesia alone (F(1,41) = 15.01; p < 0.01) and post-hoc comparisons indicated bladders of zymosan-treated rats were significantly increased in both diestrus (p < 0.01) and proestrus (p < 0.01) relative to anesthesia controls. To calculate bladder content of CGRP and SP, the concentration of peptide in each sample, measured using ELISA, was multiplied by the weight of each bladder. Therefore, bladder content of CGRP and SP was proportional to bladder weight, and increased bladder weights were associated with increased CGRP and SP levels. However, neither CGRP nor SP was significantly increased during diestrus, when bladder weights were significantly increased in inflamed rats relative to control rats. These data indicate that while bladder weight increases may be a necessary condition for increased CGRP and SP levels, bladder weight alone is not a sufficient condition to produce these increases.

### Estradiol and progesterone

The top panel of Figure 2 shows that zymosan-treated rats had consistently lower levels of estradiol compared to anesthesia-alone controls. This lowering was significant when data were collapsed across all phases of estrous (F(1,30) = 4.52; p = 0.04) but not when data were compared during any specific phase of estrous. The middle panel of Figure 2 shows that progesterone levels also tended to be reduced by zymosan but no significant reductions were found. In general, the data suggest that inflammation and/or anesthesia-alone produced some disruption in the normal levels of these hormones that would be expected on the basis of other reports that have examined levels of these hormones in normal cycling naive rats, (e.g., [21]), but as noted previously, classification was done on the basis of vaginal lavage at the time of testing.

### EIL bladder inflammation

#### CGRP and SP

**Figure 3:**
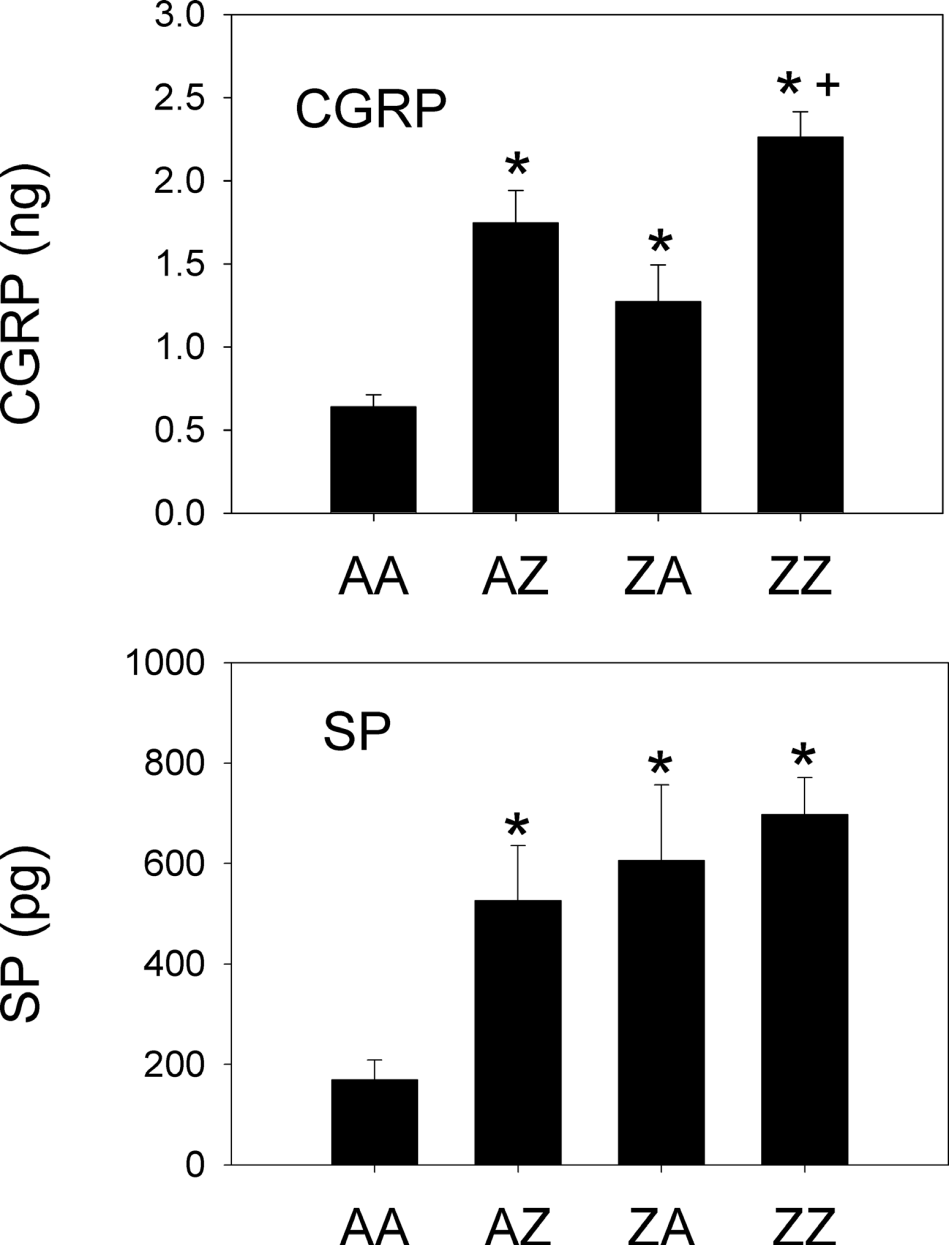
**Figure 3 presents group mean bladder content of CGRP (ng) and SP (pg) in rats treated EIL**. Error bars depict standard error of the mean (SEM). Rats were treated with either anesthesia both EIL and as adults (AA), anesthesia EIL and zymosan as adults (AZ), zymosan EIL and anesthesia as adults (ZA), or zymosan both EIL and as adults (ZZ) (n = 4-7 rats/group). Groups AZ, ZA, and ZZ differed significantly from group AA for both CGRP and SP (* significantly different from anesthesia control p < 0.05). CGRP for group ZZ also was significantly increased relative to ZA animals (+ p < 0.01).

Bladder content of CGRP in EIL-treated animals is shown in the upper panel of Figure 3 from Experiment 2. An overall ANOVA revealed a significant effect of intravesical treatment on bladder content of CGRP (F(3,19) = 19.01, p < 0.01). Follow-up comparisons revealed that all treatments significantly increased bladder content of CGRP compared to AA controls. Specifically, bladder content of CGRP in AZ (p < 0.01), ZA (p = 0.01), and ZZ (p < 0.01) animals was significantly greater than that of AA controls. Also, re-inflammation as an adult significantly increased bladder content of CGRP since bladder content of CGRP was significantly greater in ZZ animals compared to ZA animals (p < 0.01). There was a trend indicating that prior EIL inflammation may have increased bladder content of CGRP in animals re-inflamed as adults since bladder content of CGRP was also greater in ZZ animals compared to AZ animals (p = 0.03), but this comparison was non-significant after correcting for multiple comparisons.

An overall ANOVA revealed a significant effect of intravesical treatment group on bladder content of SP (F(3,19) = 5.78, p < 0.01). Follow-up comparisons revealed that all treatments significantly increased bladder content of SP compared to AA controls. Specifically, bladder content of SP in AZ, ZA, and ZZ animals was significantly greater than that of AA controls (all p's < 0.01).

The absolute levels of CGRP and SP differed between Experiments 1 and 2, but groups did not receive identical treatments in these experiments. Further, such differences may reflect different housing conditions encountered by these animals during maturation. Animals in Experiment 1 were acquired from Harlan at 12 weeks of age, while animals in Experiment 2 were born and raised in our animal housing facilities.

### Bladder Weights

Bladder weights for Experiment 2 are shown in Table 1. An overall ANOVA revealed a significant effect of intravesical treatment group on bladder weights (F(3,35) = 12.18, p < 0.01). Post-hoc comparisons indicated that all groups receiving zymosan had significantly greater bladder weights than group AA as would be expected to occur following inflammation (all p's < 0.03). Interestingly, the data from group ZA indicate that an EIL exposure to bladder inflammation alone is a sufficient condition to produce a permanent increase in bladder weight that lasts into adulthood. Also, the increase produced by EIL and adult treatment combined (group ZZ) was greater than that produced by EIL inflammation (group ZA; p < 0.01) or adult inflammation alone (group AZ; p = 0.01).

**Table 1:** Group mean bladder weights (mg ± SEM) for the groups of Experiment 2

Group	AA	AZ	ZA	ZZ
Bladder Weight	69.0 ± 13^2^	114.0 ± 2^12^	98.5 ± 13^12^	145.5 ± 9^1^

Rats were treated with either anesthesia both EIL and as adults (AA), anesthesia EIL and zymosan as adults (AZ), zymosan EIL and anesthesia as adults (ZA), or zymosan both EIL and as adults (ZZ) (n = 4-7 rats/group). Bladder weights were significantly greater in AZ, ZA, and ZZ animals compared to AA controls. In addition, the bladders of ZZ animals were also significantly heavier than those of AZ and ZA animals.

^1 ^significantly different from AA (p < 0.05)

^2 ^significantly different from ZZ (p < 0.05)

## Discussion

### The effect of estrous cycle on bladder content of CGRP and SP after acute adult bladder inflammation

Pain arising from bladder disorders is a common problem in women and some of these pain disorders are hypothesized to result from increases in the content of CGRP and SP in the bladder with consequent sensitization of bladder afferents containing these neuropeptides. Double-labeling studies in human bladder indicate that approximately 26% of CGRP-immunoreactive fibers contain SP [8,22]. In rat, approximately 40-54% of L6-S1 DRG bladder afferent cells express CGRP, whereas only approximately 2-3% of L6-S1 DRG bladder afferent cells express SP-IR, and virtually all SP-IR fibers contain CGRP [1], see also [23]. In male rats, CGRP- and SP-IR are increased in the DRG of bladder afferent neurons and the spinal cord following acute bladder inflammation with mustard oil [8]. A similar outcome has been obtained in female rats using chronic bladder inflammation with CYP, which significantly increases CGRP-IR in the L6 DRG, but not the S1 DRG. Increases in SP-containing nerve fibers in the submucosal region [24] and increased levels of NK1 receptor encoding mRNA in the vascular endothelium [23] also have been reported in biopsies obtained from IC patients. None of the above studies, however, have systematically examined influences of the estrous or menstrual cycles on measures of CGRP and SP or other factors such as EIL experience with bladder inflammation, which could serve as important contributing factors in exacerbating pain.

Experiment 1 of the present studies examined bladder content of CGRP and SP during each phase of the estrous cycle in acutely-inflamed adult rats. Both CGRP and SP were significantly increased after inflammation during the proestrus phase of estrous when compared to estrous phase-matched anesthesia-alone controls. Similar matched comparisons in the other phases of estrous were not significant although the bladder content of both CGRP and SP was significantly increased when data from all acutely-inflamed rats were collapsed across all phases of the estrous cycle. These data support the view that if CGRP and/or SP mediate bladder hypersensitivity then hypersensitivity is likely to be maximal during proestrus in bladder-inflamed animals. These data correspond to findings by other investigators showing that response thresholds for the VMR to UBD were lowest during proestrus [25] and mean response thresholds of pelvic mechanoreceptors innervating the bladder and mean bladder compliance are lowest in proestrus in adult female urethane-anesthetized rats [26]. These data also correspond reasonably well to recently reported data showing that adult female rats with zymosan-induced bladder inflammation had the most robust VMRs to UBD during metestrus and proestrus [6]. In the present study, we did not observe significant increases in these peptides during metestrus.

We observed no significant differences in CGRP and SP levels as a function of the phase of estrous cycle in rats that did not have inflamed bladders. This outcome bears similarity to a previous study that found no significant differences in the magnitude of the VMR to UBD as a function of the phase of estrous cycle in adult female rats with non-inflamed bladders [6] and are analogous to the lack of estrous cycle effects observed by Johnson and Berkley [7] in female rats with non-inflamed bladders undergoing analyses of micturition response thresholds in cystometrogram (CMG) testing. Therefore, the effects of estrous cycle are most clearly manifested under conditions of bladder inflammation.

### Bladder content of CGRP and SP in animals with or without EIL bladder inflammation and adult re-inflammation

Experiment 2 examined bladder content of CGRP and SP after an EIL exposure to bladder inflammation. EIL inflammation produced an increase in CGRP and SP bladder content that lasted into adulthood and was comparable to that produced by an acute, adult exposure to bladder inflammation (compare groups ZA and group AZ in Figure 3). Since increased bladder weights were associated with increases in CGRP and SP in both experiments, we evaluated the extent to which the increases in CGRP and SP were dependent on increased bladder weight. While increased bladder weights may well have contributed to the increased levels of these bladder peptides in both Experiments 1 and 2, two points should be noted. First, if bladder weights are critical for increasing the levels of these peptides then they are only a necessary condition and not a sufficient condition for this to occur. That is, significant bladder weight increases were observed in rats with inflamed bladders during diestrus in Experiment 1, but we observed no significant increases in CGRP and SP levels during diestrus. Second, even when we controlled for the increased bladders weights of inflamed rats examined in Experiment 2, by examining the content per mg of bladder tissue, we still observed significant increases in CGRP and SP in groups AZ, ZA, and ZZ relative to group AA.

The issue of bladder weight is important, however, because heavier and presumably larger bladders containing more neuropeptides in the absence of other changes in the nervous system may functionally translate into greater input into the spinal cord and enhanced bladder sensitivity. At a minimum, these data support the view that an EIL experience with bladder inflammation may potentially predispose an organism to experience a painful bladder disorder as an adult if it is mediated by CGRP and/or SP especially when followed by re-exposure to bladder inflammation as an adult.

Some studies suggest that CGRP and SP act synergistically [27-30] due to CGRP inhibition of an endopeptidase which degrades SP [31]. Thus, SP may be the primary neurotransmitter that enhances spinal nociceptive transmission under conditions of bladder inflammation during proestrus and CGRP may act as a neuromodulator serving to facilitate the spinal activity of SP at this time. One must also entertain the possibility that similar interactions between CGRP and SP could occur peripherally in the bladder to influence bladder afferent sensitivity in some manner.

### Gonadal Hormones

In studies of acute, adult inflammation (Experiment 1), we observed that bladder inflammation generally reduced estradiol levels during all phases of estrous and the levels were significantly lower than those of anesthesia-alone controls when collapsed across all phases of estrous. Progesterone levels also tended to be reduced by bladder inflammation but these reductions were not statistically significant. Finally, serum hormone levels indicated disruption in normal gonadal cycling due to inflammation and/or isoflurane anesthesia despite the fact that animals showed normal vaginal lavages and were therefore classified solely on that basis.

Evidence concerning whether estrogen and/or progesterone either increase or decrease pain sensitivity is controversial. There is evidence for antinociceptive effects of estrogen in somatic pain models involving hot plate and tail withdrawal responses [11], phase 2 of the formalin test [32,33], and in slow developing mechanical and thermal hyperalgesia of the abdominal and pelvic muscles produced by ovariectomy (OVX) [34]. However, the opposite outcome has been observed in other studies of visceral nociceptive systems. In these studies, OVX reduced VMRs to UBD [35] and to colorectal distension [36] and the acute replacement of estrogen in these model systems produced increases in VMRs [37,38]. In mice, the presence of estrogen receptor-alpha effects in the absence of estrogen receptor-beta effects results in histological alterations of the bladder reminiscent of human IC patients [39]. There may be tissue-specific differences in estrogen effects, perhaps involving differential activation of estrogen receptor-alpha and beta that contribute to contrasting pro- and anti-nociceptive effects in different tissues.

Interestingly, we have previously observed that rapidly falling levels of estrogen are associated with an augmentation of the VMRs [38]. Therefore a sudden change in circulating estrogen, rather than a sustained increase (as during prolonged hormone replacement) or decrease (as during testing several days after OVX), may lead to increased responses to visceral nociceptive stimuli. In the context of the present experiment, and using similar parameters, it is notable that inflammation resulted in a general drop in estrogen levels. A drop in estrogen perhaps could contribute to the augmentation of VMRs observed in other studies and also could account for the general tendency to observe increases in CGRP and SP levels across all phases of the estrous cycle in the present study by promoting CGRP and SP synthesis.

### Clinical Relevance

The clinical relevance of these basic science findings and our EIL animal model relate to chronic pain disorders, such as PBS/IC. As noted in previous studies, c.f. [15] this EIL animal treatment results in increased micturition frequency, bladder hypersensivity, and decreased intercontraction intervals during CMG testing which parallel human PBS/IC symptoms of increased urinary frequency, bladder pain, and urgency. Our EIL animal model also has been shown to mimic outcomes of a human clinical study by Mukerji et al. [40] of PBS/IC patients. They found that 76.5% of female patients with PBS/IC reported pain during an intravesical instillation of ice cold saline whereas females with idiopathic detrusor overactivity (IDO), neurogenic detrusor overactivity (NDO), or normal controls did not report pain during similar tests. As a form of reverse translational research to test the validity of our EIL model, we evaluated whether a similar cold sensitivity was present in our rodent model system. When tested as adults, EIL bladder inflammation resulted in significantly increased VMRs to intravesical cold stimuli even in the absence of re-inflammation as an adult [41].

## Conclusions

The present studies demonstrate that CGRP and SP are increased by adult bladder inflammation, and that EIL exposure to bladder inflammation alone can also produce an increase in CGRP and SP lasting into adulthood. Phase of the estrous cycle also impacts bladder CGRP and SP content; CGRP and SP are maximally increased during the proestrus phase of the estrous cycle in inflamed adult female rats. Increases in endogenous CGRP and SP may translate into increased spinal nociceptive transmission and enhanced bladder sensitivity. Other studies from our laboratory also have shown that descending inhibitory [6,14] and facilitatory [42] pain modulatory systems are altered by bladder inflammation, indicating that complex changes in pain processing may occur at multiple points and these changes may interact to produce chronic bladder pain. The clinical relevance of this study includes the suggestion that EIL experience with bladder inflammation may be one of many possible factors that could predispose an individual to experience a painful bladder disorder as an adult by increasing primary afferent content of CGRP and/or SP. In addition, this study suggests a possible physiological basis for alterations in bladder sensitivity during the menstrual cycle.

## Abbreviations (in order of appearance)

AA: Anesthesia (Early-in-life) Anesthesia (Adult); ANOVA: Analysis of variance; AZ: Anesthesia (Early-in-life) Zymosan (Adult); CGRP: Calcitonin gene-related peptide; CMG: Cystometrogram; CYP: Cyclophosphamide; DRG: Dorsal root ganglia; EIL: Early-in-life; ELISA: Enzyme-linked immunosorbent assay; Group A: Anesthesia; Group Z: Zymosan; IASP: International Association for the Study of Pain; IC: Interstitial cystitis; IDO: Idiopathic detrusor overactivity; mRNA: Messenger ribonucleic acid; NDO: Neuropathic detrusor overactivity; NK1: Neurokinin 1; OVX: Ovariectomy; PBS/IC: Painful bladder syndrome/interstitial cystitis; RIA: Radioimmunoassay; s.c.: Subcutaneous; SEM: Standard error of the mean; SP: Substance P; UBD: Urinary bladder distension; VMR: Visceromotor reflex; ZA: Zymosan (Early-in-life) Anesthesia (Adult); ZZ: Zymosan (Early-in-life) Zymosan (Adult)

## Competing interests

The authors declare that they have no competing interests.

## Authors' contributions

AR, ADS, and TJN conceived of the study, and participated in its design and coordination. ADS, CLB, and MTR were involved in bladder tissue collection. TJN, MTR, and CLB performed and analyzed the results of ELISAs. AR and ADS performed the statistical analyses. ADS and AR drafted the manuscript, and all authors were involved in revisions. All authors read and approved the final manuscript.

## Pre-publication history

The pre-publication history for this paper can be accessed here:

http://www.biomedcentral.com/1471-2490/11/18/prepub
